# Antimicrobial use in the pandemic

**DOI:** 10.2471/BLT.22.020522

**Published:** 2022-05-01

## Abstract

Antimicrobial misuse has risen sharply during the pandemic, exacerbating already worrying trends. Lynn Eaton reports.

When the first patients with coronavirus disease 2019 (COVID-19) started coming into Kenyatta National Hospital in Nairobi, Kenya in March 2020, emergency room staff were unsure how to respond.

“When the pandemic started, the treatment guidance just didn’t exist,” says Dr Loice Achieng Ombajo, an infectious disease specialist at the University of Nairobi who was in the front line of the hospital’s response.

Adding to the stress this created were the circumstances in which patients were being admitted. Despite being a leading teaching hospital, Kenyatta National only had 10 critical care beds with a ventilator, and yet patients kept coming in, often in acute respiratory distress.

Desperate to do something, doctors treated patients with broad spectrum antibiotics. “Even though they knew this was a viral infection, they went ahead and treated people as if they had community-acquired pneumonia. We had arguments about it on the wards,” Ombajo says.

The doctors in Nairobi were not alone.

“The lack of COVID-19 treatment guidelines in the initial stages of the pandemic meant that doctors didn’t know what to do, and one of the typical default responses was to prescribe antibiotics,” says Dr Lo Fo Wong Danilo, regional adviser of the World Health Organization’s (WHO) Control of Antimicrobial Resistance Programme for Europe.

The extent to which more antibiotics were prescribed than needed has yet to be fully described since surveillance of antibiotic use was among many essential health activities to be deprioritized during the pandemic, but early indications suggest that it was significant.

For example, a study published in the March 2021 issue of *Clinical infectious diseases* which randomly sampled a cohort of 1705 patients hospitalized with COVID-19 in 38 hospitals in the state of Michigan in the United States of America between March and June 2020 reported that, while only 3.5% (59/1705) had a confirmed bacterial infection, an average 56.6% (965/1705) were prescribed antibiotics. Other studies and multiple-study surveys have reported similar findings.

Danilo points out that the overprescribing of antibiotics did not begin with the pandemic. “COVID-19 made a bad situation worse,” he says, but he cautions against oversimplifying what is in fact a complex emerging picture.

“Changes in the ecology of resistance mechanisms have been observed across the region.”

Pilar Ramon-Pardo

That picture includes divergent trends within Europe where, according to the latest data from the European Centre for Disease Prevention and Control, between 2019 and 2020, there was an overall 17.6% decrease in antibiotic use in 29 countries (27 European Union plus Iceland and Norway) from 19.9 defined daily dose (DDD) per 1000 people per day to 16.4 DDD per 1000 in 2020. Most of this decrease occurred in the primary care sector and possibly resulted from a decrease in the number of primary care consultations due to factors that may include patient hesitancy, difficulties in obtaining an appointment, and lower incidence of respiratory tract infections in 2020.

However, according to Danilo, in many of the 24 other countries in the WHO European Region, especially those where over-the-counter sales of antibiotics are poorly regulated, antibiotic prescription appears to have risen sharply.

A sharp rise in antibiotic use is also being observed in Latin America and the Caribbean according to Dr Pilar Ramon-Pardo, regional adviser with the Pan American Health Organization (PAHO) which has been collating information on antimicrobial use in the region in support of antimicrobial stewardship (optimized use and preservation) measures.

“We have seen a tremendous increase in the use of broad-spectrum antibiotics, with almost all hospitalized COVID-19 patients being given an antimicrobial as part of their treatment, while only around 7% have a secondary infection that would justify the use of such drugs,” Ramon-Pardo says.

Ramon-Pardo also notes increased use of the antimalarial hydroxychloroquine and the antiparasitic ivermectin, both in clinical settings and in the community, which has continued despite the lack of evidence regarding their efficacy as COVID-19 treatments.

What impact the spike is having on Latin America’s microbes is still unclear, but Ramon-Pardo is concerned. “Changes in the ecology of resistance mechanisms have been observed across the region,” he says. These changes include an increase in the geographical distribution of enzymes that can break down carbapenems, a class of antibiotics typically reserved as a last resort for multidrug-resistant infections.

In October 2021, PAHO flagged reports from the national reference laboratories of several countries in the region on the emergence of carbapenem-resistant *Enterobacterales*, an order that includes pathogenic species, such as *Escherichia coli* and *Salmonella enterica*.

In the light of those findings, PAHO recommended that all Member States implement and strengthen epidemiological surveillance and investigation to detect and characterize resistance mechanisms to carbapenems and to support the implementation of measures to prevent transmission in health facilities, as well as to effectively implement programmes to optimize the use of antibiotics.

“Responsible and appropriate use of antibiotics is clearly key to ensuring that they continue to be effective,” says Dr Haileyesus Getahun, Director of WHO’s Department of Antimicrobial Resistance Global Coordination, drawing attention to WHO guidance on COVID-19 treatment options – including antibiotics – that was first published by WHO in January 2021 and has since been regularly updated.

WHO’s efforts to tackle antibiotic misuse predate the pandemic, notable initiatives including the launch of the AWaRe campaign in 2017 to guide antimicrobial prescription and treatment while monitoring consumption. AWaRe breaks down antibiotics into three groups, labelled “access”, “watch” and “reserve”, the first comprising the antibiotics of choice for the 25 most common infections, the second the antibiotics recommended only for specific, limited indications and the third antibiotics that should only be used as a last resort.

WHO is also supporting countries in setting up and strengthening national antimicrobial resistance surveillance through the Global Antimicrobial Resistance and Use Surveillance System and improving diagnostic capacity to ensure that infections are identified, laboratory confirmed and properly treated. Notable developments in this regard include the establishment of the Central Asian and European Surveillance of Antimicrobial Resistance network and the Latin American and Caribbean Antimicrobial Resistance Monitoring Network.

Despite such efforts, antibiotics continue to be overprescribed in many settings, a fact that has been brought home in the pandemic and is a source of frustration for many experts, including Getahun. “Non-adherence to recommended protocols is a problem and we need a better understanding of what is driving it,” he says.

To that end WHO has launched a Tailoring Antimicrobial Resistance Programmes initiative that is drawing on behavioural science to support activities including the monitoring of public knowledge, risk perceptions, behaviours and trust. The initiative also supports behaviour-change campaigns targeting specific audiences.

“COVID-19 made a bad situation worse.”

Lo Fo Wong Danilo

PAHO is also focusing resources on improving practice, with efforts that include the launch of a pilot in Paraguay to monitor antibiotic use in clinical settings. “Currently, there is no country in the region that is capturing that kind of information in any detail,” says Ramon-Pardo, who is hopeful that the pilot can serve as a model for other countries.

Dr Anahi Cristina Dreser Mansilla, a researcher at the National Institute of Public Health in Cuernavaca, Mexico, thinks that one of the reasons for lack of adherence to recommended protocols may be a lack of policy follow-through. “Mexico only introduced a national policy on antibiotic use in 2018 and once it was published, nothing happened. There was no strategy to communicate the guidelines to physicians.”

According to Dreser, the result was a low level of adoption at the clinical level. “Prior to the pandemic, only half of the main hospitals in Mexico had an antimicrobial policy,” she says. Dreser worked with PAHO on a project to boost policy uptake but the project was halted because of the pandemic. “To embed anything during the pandemic was unthinkable,” she says. “We kept trying to work with hospitals, but they would say they just couldn’t. They were too overwhelmed.”

In the last few months Dreser and a group of colleagues have introduced training in 15 hospitals in Mexico on antimicrobial stewardship and monitoring consumption. She has also helped to organize some sessions for doctors on antibiotic and antimicrobial use in COVID-19 patients.

Ombajo is also talking to clinicians. She held weekly two-hour seminars online during the height of Kenya’s epidemic and more than 1000 clinicians attended, presenting their COVID-19 cases and outlining the treatments they were using. “In-country physicians, virologists and intensive care unit specialists also wrote guidance that was specific for Kenya,” she says.

Because the antimicrobial misuse challenge is not limited to health-care settings (indeed the challenge is thought to be far greater outside health facilities, and the pandemic may have increased self-medication by patients unable to consult doctors redirected to COVID-19), Ombajo has also made an effort to spread the word by appearing on television shows.

“People need to realize that taking an antibiotic is not the same as taking a drug for high blood pressure. The antibiotics exert a selective pressure on the organisms they are designed to destroy or inhibit, which drives the emergence of competitive advantages. The more antibiotics are used, the more the bugs change. That doesn’t just affect you, it affects your family, your hospital, your neighbour, your environment. We have to think about that when we take them. We have to think about that more and more.”

**Figure:**
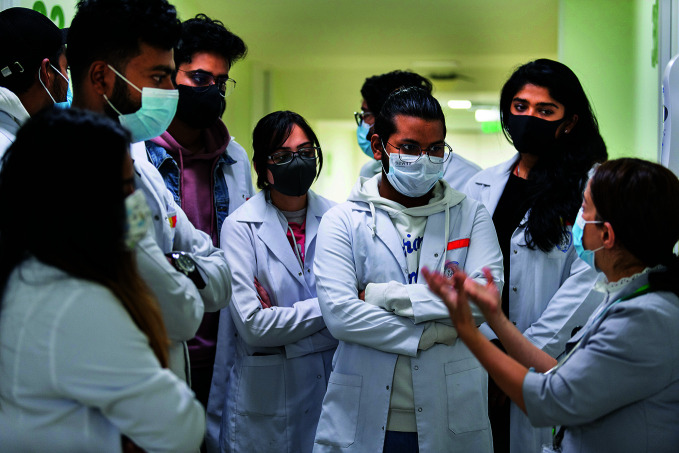
A paediatrician at Wigmore Clinic in Yerevan, Armenia, explains the importance of hand hygiene as part of infection control efforts.

WHO/Nazik Armenakyan

**Figure:**
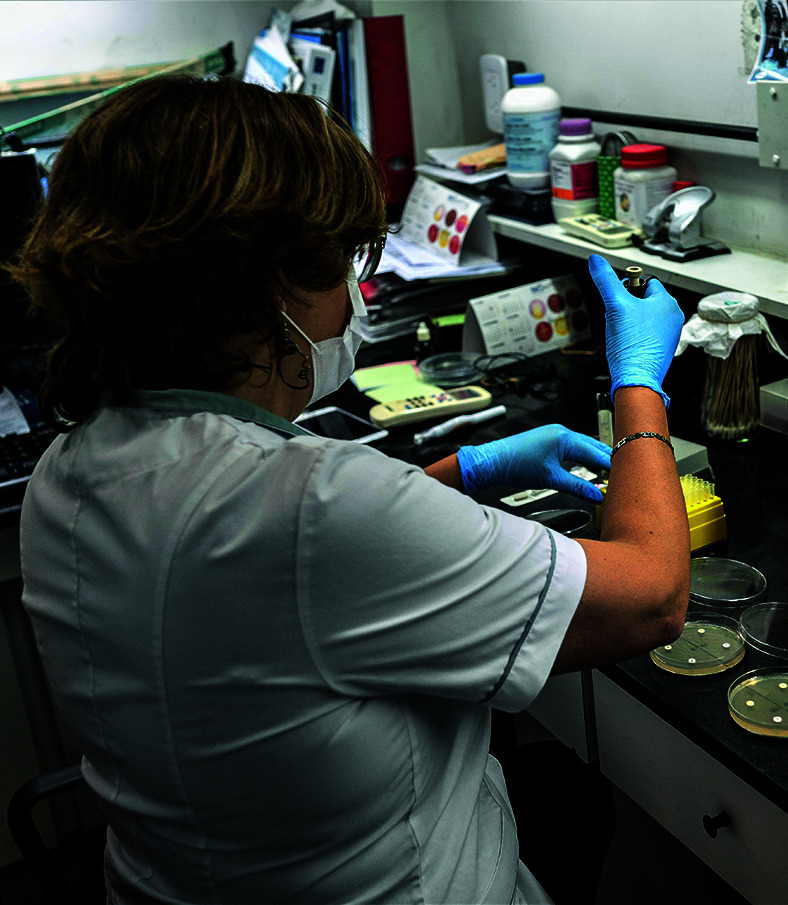
A biochemist performs multiple assays of antimicrobial susceptibility testing at Malbrán Institute in Buenos Aires, Argentina.

WHO/Sarah Pabst

